# Dual-Electrode Glass Ribbons Intended for Use in Microplasma-Based Sensors

**DOI:** 10.3390/s25226814

**Published:** 2025-11-07

**Authors:** Mathieu Bonnardel, Angeline Poulon-Quintin, Sylvain Danto, Bruno Bousquet, Lionel Teulé-Gay, Thierry Cardinal

**Affiliations:** Institut de Chimie de la Matière Condensée de Bordeaux, Unité Mixte de Recherche 5026, Université de Bordeaux, Centre National de la Recherche Scientifique, Institut Polytechnique de Bordeaux, F-33600 Pessac, France; mathieu.bonnardel@u-bordeaux.fr (M.B.); sylvain.danto@icmcb.cnrs.fr (S.D.); bruno.bousquet@icmcb.cnrs.fr (B.B.); lionel.teule-gay@icmcb.cnrs.fr (L.T.-G.); thierry.cardinal@icmcb.cnrs.fr (T.C.)

**Keywords:** multimaterial fiber/ribbon, stack-and-draw, gas sensor, optical emission analyses of plasma discharge

## Abstract

**Highlights:**

**What are the main findings?**

- Long multimaterial ribbons composed of soda–lime silicate glass and AA 2024 electrodes were successfully developed via the S&D technique for the first time.
- The in situ formation of an oxide layer on the tip of each electrode drastically increased the duration of the plasma emission without the need for additional post-functionalization of the electrode surfaces but required adapted interspacing values.

**What is the implication of the main finding?**

- The control of the inter-electrode spacing value is critical to improving the microplasma emission time when the ribbon is used as miniaturized gas detector in hydrocarbon emission detection.
- Using an aluminum alloy to make the electrodes can greatly increase the distance between the analyzed region and the location of the measuring and electricity generation devices.

**Abstract:**

The combination of microplasma generation and optical multi-material fiber technologies enables real-time diagnostics. The stack-and-draw technique has emerged as a promising method for creating multimaterial fibers suitable for plasma-based diagnostics. The elaboration of such devices for the generation of long-lasting microplasma for real-time and remote analyses remains challenging due to the difficulties of reaching long lengths without defects and with continuous electrodes. Post-functionalization of the electrode surface is also required to increase the plasma emission duration. In this study, glass was preferred over polymers for producing rectangular fibers (ribbons) that are easy to stack without wasting space and are resistant to high operating temperatures. Conversely, an aluminum alloy was chosen for the electrodes to reduce discontinuity defects. With the chosen bi-electrode geometry, the cooling rate during drawing has to remain between 200 and 300 °C/s to limit defect formation and guarantee low electrical resistivity. During plasma generation, an in situ oxide layer forms on the tip of each electrode. This results in a significant increase in plasma emission duration without the need for an additional post-functionalization step after drawing. These ribbons were tested in combination with an optical emission spectrometer to create a miniature gas detector for hydrocarbons.

## 1. Introduction

In recent years, new approaches to materials processing have enabled the manufacturing of multimaterial fibers. Due to their ability to incorporate multiple functionalities into a singular platform, these innovative fibers are in high demand across various fields, such as health care, energy harvesting, and production of sensors and wearable textiles [1,2,3,4,5,6]. One of the newest topics in the field is the potential combination of microplasma generation and optical multimaterial fiber technologies [7,8,9,10], which opens possibilities for real-time diagnostics in remote, confined, and/or high-temperature environments and/or under various pressure conditions (atmospheric or low vacuum [11,12]). Several authors have successfully created devices based on multimaterial fibers capable of generating electrically induced microplasma discharges. One approach involves modifying pre-existing empty-core optical fibers with a conductive paste and then injecting a gas or a mixture of a gas and a precursor into the empty cores of the fibers in order to generate a microplasma plume or jet at the fiber tip [11,13,14,15]. Another approach consists of creating the device in an all-in-one step, either by inserting the electrodes in the optical fibers after drawing or by inserting the metallic electrodes into an amorphous clad preform, which is then co-drawn into fibers [16,17]. The stack-and-draw (S&D) technique has emerged as a promising method for integrating metallic electrodes with precise geometries within the fiber preform, enabling the creation of complex multimaterial structures with predefined electrode locations. This technique has been shown to enhance the control over electrode positioning, making it suitable for applications requiring high precision, such as plasma-based diagnostics and sensing [2,3,4,18,19,20]. It is possible to design fibers with embedded metal electrodes by confining and controlling the crystalline material flow in an amorphous support compatible with the drawing [17,18,19,20,21,22]. Co-drawing of the two materials allows fibers with glass cores and pure metal electrodes to be prepared over longer lengths [23,24].

The elaboration of such a device remains challenging, especially when the aim is the generation of a long-lasting microplasma for real-time and remote analyses. Working under atmospheric pressure requires the application of a high voltage bias between the metallic electrodes to induce the electrical breakdown of the gas [25]. These process conditions result in electrode joule heating. The generated plasma evaporates/sputters both the cladding and the metallic electrodes if no post-functionalization of the metallic electrodes occurs [7,26]. Some authors used a semi-insulating layer (ρ~10^6^ Ω.cm) coating at the tips of pure tin electrodes embedded in a polymer cladding [7]. The consequences are a decrease in the electronic density in the gas gap, which prevents the transition from a glow discharge to an electrical arc. The localization and stability of the plasma at the electrode tip is improved as the plasma generation duration increases [7]. The use of such a device is limited to low-temperature environments due to the polymer cladding glass transition temperature (T_g_ = 158–228 °C for PES). Unfortunately, obtaining long fibers with the co-drawing of glass and pure metals remains a real challenge due to the appearance of discontinuities in the metallic electrodes during the thermal drawing [20,26]. Some authors have shown the benefit of choosing a metal with high melting temperature and low density (pure aluminum: T_m_ (Al) = 658 °C, d (Al) = 2.7 g/cm^3^) for increasing the control over the drawing step [8,18].

Long glass–metal ribbon fibers were produced in this work. The continuity of metallic electrodes with low electrical resistivity was preserved with appropriate material selection and fine control over process parameters. A key innovation compared to previous works lies first in the use of an aluminum alloy rather than pure aluminum as the electrode material. The aim is to preserve electrical properties when fiber length increases, avoiding the formation of discontinuities. Drawing ribbons helps to preserve the preform’s shape by reducing its dimensions in a controlled way. The ability to generate microplasma at the tip of the bi-electrode fibers to analyze various gases is demonstrated. To our knowledge, the aging of this device during plasma discharge is analyzed for the first time. The novelty of our approach, compared to existing approaches in the literature, also derives from the formation of an efficient in situ dielectric layer on the electrode tips during plasma generation.

## 2. Materials and Methods

### 2.1. Materials and S&D Process

**Figure 1:**
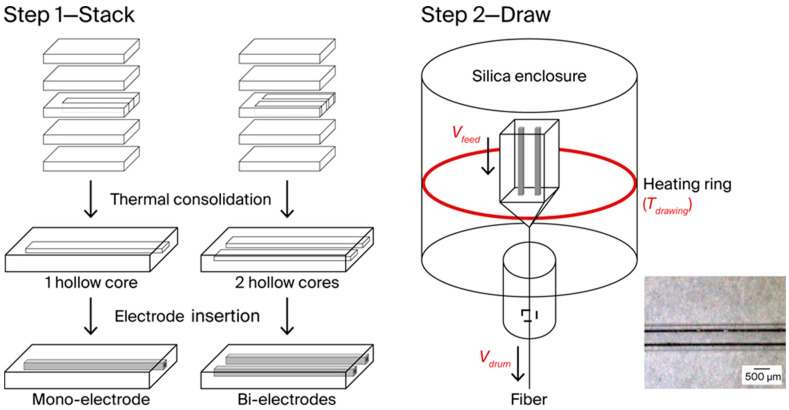
Schematic representation of the S&D process scenario in two steps: (1) stacking of the preform with the insertion of one or two electrodes and (2) drawing. The key controlled process parameters are highlighted in red. An example of a bi-electrode fiber is shown in the bottom-right corner (top view, electrodes in dark).

Before the multifiber drawing, a macroscopic object called a preform is drawn into a centimeter-scale version of itself and fabricated from glass parts. This preform is used as a support for the metallic electrodes (Figure 1, step 1). Regarding material selection, the melting temperature (T_M_) of the metal used for electrodes has to be lower than the drawing temperature (T_drawing_, Figure 1, step 2). T_drawing_ usually corresponds to a temperature at which the viscosity (η) of the glass is between 10^4^ and 10^6.5^ Pa.s [27].

The AA 2024 aluminum alloy (Table 1, Alcan Company, Alma, QC, Canada) was chosen based on the following:

- Good electrical properties. At equal weight, aluminum has twice the electrical conductivity of copper, hence its applications in long-distance, high-voltage electricity transmission. Even though the electrical resistivity of aluminum alloys is higher than for pure aluminum (around +40%), an aluminum wire conducts 2.1 times more electricity than a copper wire and 18 times more than a steel wire of the same weight.
- Light weight and better mechanical properties than pure aluminum even at high temperature [28].
- Low cost. For the same electrical conduction, the price of an aluminum alloy conductor is equal to 40% of the price of a copper conductor (data Euralliage^®^, Gonesse, France). The AA 2024 alloy price is similar to that of 99.9% purity aluminum (2.98 USD/kg for square bars compared to around 2.6 USD/kg for pure aluminum plates).

Moreover, the melting temperature and density of AA 2024 alloy are similar to those reported for pure aluminum.

Microscope glass slides purchased from Agar Scientific (reference G251P, Agar Scientific, Rotherham, UK) were selected in line with the thermal drawing protocol (*T_M_* (electrode metal) <<< *T_x_* (glass)) and *T_M_* (electrode metal) << *T_drawing_* (glass)). They were chosen for their high glass transition (*T_g_*) and crystallization onset (*T_x_*) temperatures. This provides a high thermal working range (ΔT = *T_x_* − *T_g_*) to prevent crystallization during thermal drawing, as discussed by Schmidt [17].

Figure 1 presents the overall S&D process scenario. First (Figure 1, step 1), glass slabs are cut (76 × 26 × 1.2–1.3 mm^3^), polished and washed with ethanol before being stacked into a rectangular preform (76 × 26 mm^2^ × (5–7) mm thick). Some inner glass slabs are recut to create hollow spaces (40 × 1 × 1 mm^3^) inside the preform for the insertion of the alloy rods after a step involving thermal consolidation at 612 °C (*T_g_* + 50 °C) for 3 h. Then, the glass stack is annealed at 542 °C (*T_g_* − 20 °C) for 12 h. During heat treatment, di-silicate bridges (Si–O–Si) are formed between the Si–OH groups present on the surface of the glass slides to bond the different glass slabs of the stack together [8,29]. After cooling, AA 2024 rods (40 × 1 × 1 mm^3^) are inserted. The preform is ready to be fixed to a silica rod before being introduced inside the silica chamber of the drawing tower.

Thermal stretching is performed using a fiber-drawing tower equipped with an induction-heating furnace. A graphite-heating element is heated in a silica chamber under Argon (3 N) flow of 0.8 L.min^−1^. The heating ramp (10 °C.min^−1^) starts when the temperature reaches 350 °C all the way up to the T_drawing_ of the preform. Then, the thermal drawing step (Figure 1, step 2) can start. Key process parameters are as follows: (1) T_drawing_ (780–820 °C in this study), which impacts the viscosity of the glass cladding and the cooling rate (CR); (2) V_feed_ (2 mm.min^−1^ in this study), which monitors how the preform descends into the heating zone, i.e., the quantity of matter drawn per minute; and (3) V_drum_ (2–13 m.min^−1^ in this study), which impacts the overall drawing tension on the multimaterial fiber as it is wrapped around the drum. The corresponding applied charge must stay at around 100 g to maintain a sufficient interface tension between the glass and the metal so that the stretch remains homothetic.

The CR (°C.min^−1^) is estimated using the variation in temperature (ΔT) as a function of time (Δt), itself related to D, the distance between the hotspot of the drawing tower and its lower opening at room temperature (D = 0.2 m) according to Equation (1):

(1) $$CR=\frac{∆T}{∆t}=\frac{T_{drawing}-T_{room}}{D}.V_{drum}$$

### 2.2. Characterization Techniques

A Netzsch (Selb, Germany) 404PC Differential Scanning Calorimeter (DSC) was used to assess the transition temperatures of the glass (T_g_ and T_x_) and the melting temperature T_M_ of the AA 2024 with a precision of ±2 °C. The analysis was performed on 60 mg of glass sample, 10 mg of AA 2024 and 10 mg of multimaterial glass–metal fiber. The samples were placed in a platinum crucible and heated (10 °C.min^−1^) under an Ar (3 N) flow (10 mL.min^−1^) from 200 to 1000 °C for the glass and from 20 to 675 °C for the AA 2024 alloy and the hybrid fiber.

A conventional SEM (Jeol 6360A, Jeol, Tokyo, Japan) was used to image the cross- and side-sections of the multimaterial fibers. Three fiber cross-sections were mirror-polished for each set of process parameters to verify the homothetic nature of the draw.

The electrical continuity of each electrode was verified by measuring the electrical resistance. The two ends of each electrode were connected with a multimeter using silver conductive paste. More reliable electrical resistivity measurements were conducted with the four-probe method (four copper wires fixed with silver wax to the polished electrode on their longitudinal surface) on various fibers with different drawing parameters. The fiber lengths varied from 3 to 5 cm. The distance between the central wires was measured each time (close to 1 mm). The current voltage U was measured for multiple current intensities I. To obtain the resistance R value of the AA 2024 alloy depending on the process parameters, i.e., its microstructure, Ohm’s law (U = R.I) was used. The electrical resistivity ρ (Ω.m) was calculated using the following Equation (2):

(2) $$\;\;\;\rho =\frac{R.S}{L}$$

where R (Ω) is the measured resistance, S (m^2^) is the electrode cross-section area and L (m) is the length of the tested electrode, i.e., of the multimaterial fiber. Each fiber was tested three times.

Electrically induced discharge plasma can be formed in a neutral gas if it is given enough energy from an applied electric field [30]. In this study, a high-voltage generator (Brandenburg III, Keiser Power Electronics, Denmark) was connected to both electrodes for plasma generation at the tip of a bi-electrode fiber. An optical spectrometer AvaSpec-ULS4096CL-EVO (Avantes, Apeldoorn, The Netherlands) with a Full-Width Half-Minimum (FWHM) resolution of 1.2–1.3 nm was used to record the plasma emission between 170 and 1100 nm in order to analyze the plasma emission, to detect potential degradation of the fiber and to register chemical elements that were present.

## 3. Results

### 3.1. Thermal Analysis Results

**Figure 2:**
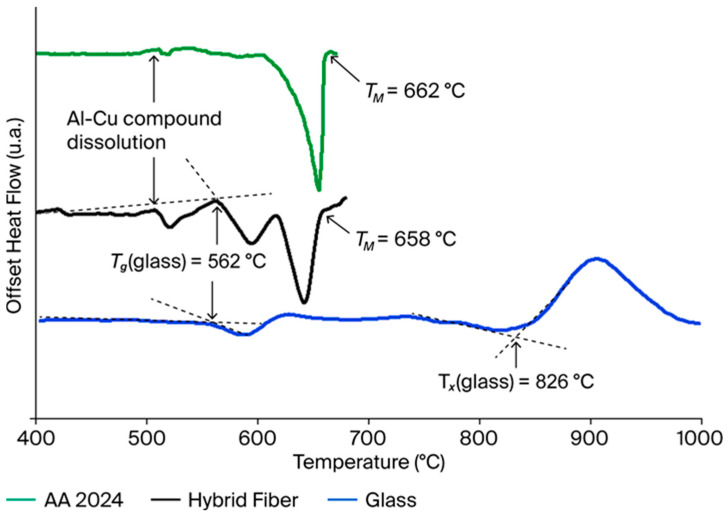
DSC analysis results: AA 2024 alloy (green curve), microscope glass slides (blue curve) and hybrid fiber (black curve).

Three thermograms are shown in Figure 2: AA 2024 (in green), microscope glass (in blue) and electrode hybrid fiber (in black). In the case of the AA 2024 alloy and the hybrid fiber, the first endothermic event occurring between 505 and 508 °C is related to the dissolution of the Al-Cu compounds/precipitates (Al_2_Cu and Al_2_CuMg) [31]. The T_g_ of the glass (both in microscope glass slides and multimaterial fibers) corresponds to the onset of the endothermic event occurring around 562 °C. The glass T_x_ relates to the onset of the exothermic crystallization event occurring at 826 °C. The AA 2024 melting is an extremely endothermic event. The T_M_ related to the endset of this event is estimated to be around 660 °C for the AA 2024 alloy and the hybrid fiber. This result indicates preservation of the AA 2024 composition during the thermal drawing.

### 3.2. Control of the Electrode Shapes and Microstructures

**Figure 3:**
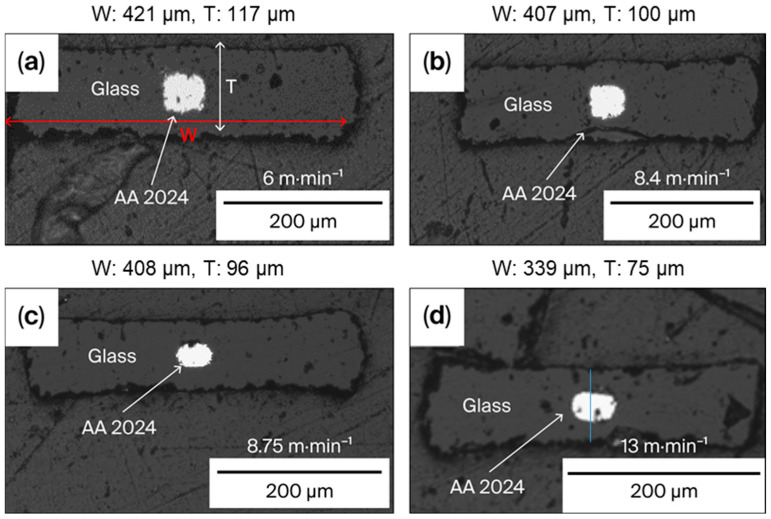
Cross-section of fibers drawn at V_drum_: (**a**) 6.0 m.min^−1^, (**b**) 8.4 m.min^−1^, (**c**) 8.75 m.min^−1^ and (**d**) 13.0 m.min^−1^.

Figure 3 shows the cross-sections of four fibers obtained with V_drum_ values of 6.0, 8.4, 8.75 and 13.0 m.min^−1^. The V_feed_ and T_drawing_ values are kept constant at 2 mm/min and 790 °C, respectively. Values of the width (W) and thickness (T) for all the fibers presented are specified. Both W and T as well as the fiber cross-section area (W × T) are inversely proportional to V_drum_. Until 8.4 m.min^−1^, the electrode shape remains squared as in the preform. A rectangular shape appears at higher values of V_drum_.

The average ratio W/T estimated on cross-section images (at least five randomly selected along the fiber) is kept constant (less than 5% difference) after drawing independently of V_drum_ at about 4.12 (±0.20) for the drawn fibers and 3.93 ± 0.09 for the preform.

**Figure 4:**
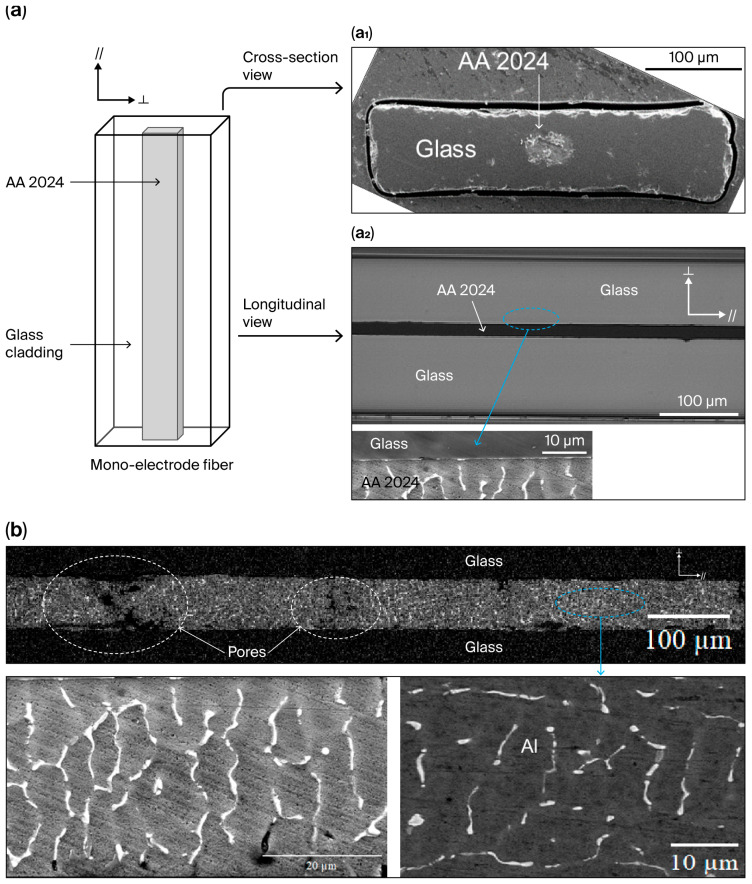
(**a**) Schematic representation of the mono-electrode fiber. Direction of the drawing axis (//) and that of the axis perpendicular (┴) are specified. (**a_1_**) SE-SEM cross-section picture of a mono-electrode fiber (390 × 88 µm^2^), T_drawing_: 790 °C, V_feed_: 2 mm.min^−1^ and V_drum_: 9 m.min^−1^. (**a_2_**) Optical microscopy longitudinal view in transmission of a piece of fiber and SEM picture of the interface between the electrode and the glass. (**b**) BSE-SEM longitudinal view of the fiber (mirror-polished). The enriched Cu-Mg aluminum phases are white, the Al phase is gray, and the pores are dark (some are circled with dotted lines). Zooming in on the area facilitates their observation.

Figure 4a is a schematic representation of a mono-electrode fiber. The parallel axis (//) to the drawing direction and the perpendicular axis (┴) are specified. Images presented in Figure 4a_1_,a_2_ help to visualize the consolidation of the glass cladding. No air bubbles or cracks are visible. The interface between the glass and the electrode is continuous (zoom, Figure 4a_2_). As noticed for similar parameters (V_drum_ = 9 m/min in Figure 4 and 8.75 m/min in Figure 3), the electrode presents a rectangular shape. The right choice of T_drawing_ maintains a high viscosity for the glass, thus preserving its shape and containing the molten alloy. BSE-SEM observation of a longitudinal section presented in Figure 4b helps to visualize the presence of pores (in dark) inside the electrode and of a second phase (in white) enriched in Cu and Mg. This presence could be detrimental to the electrode’s electrical properties.

### 3.3. Electrical Properties of the Drawn Fiber

**Figure 5:**
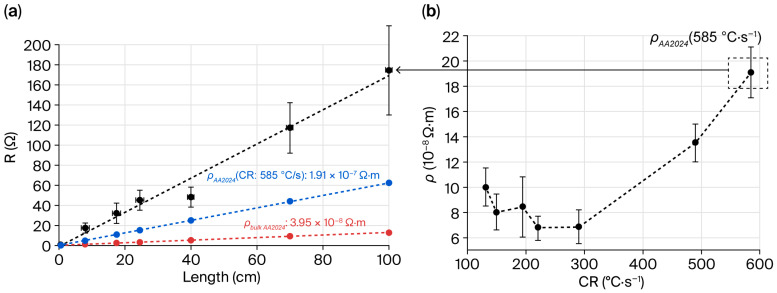
(**a**) Evolution of the electrical resistance (R) of a mono-electrode fiber (cross-section area of the fiber: 390 × 88 µm^2^, that of the electrode S: 50 × 61 µm^2^, CR: 585 °C.s^−1^) as a function of its length (in black). Theoretical evolution of the electrical resistance of a similar electrode with a theoretical resistivity similar to that of the AA 2024 alloy (ρ = 3.96 10^−8^ Ω.m, in red) or to that of the AA 2024 melted and then cooled down at 585 °C.s^−1^ (1.91 10^−7^ Ω.m, in blue). (**b**) Evolution of the electrical resistivity (ρ), depending on the CR (°C.s^−1^) for a fiber length of 5 cm.

Figure 5 presents the evolution of the electrical properties of the mono-electrode fibers: first, for a fixed CR value, as a function of the fiber length used during the measurements (Figure 5a), and second, as a function of the cooling rates (CRs), estimated from Equation (1) (Figure 5b). The cross-section surface (S) for all calculations and each fiber is assumed to be constant, regardless of the fiber length. This assumption could be made even if small variations exist (cf. Figure 4) because their impact on the resistance or resistivity values is negligible compared to the standard deviation obtained for each point, as shown in Figure 5a,b.

The evolution of the electrical resistance (R) of the mono-electrode fiber drawn at 9 m.min^−1^ (i.e., cooled down at 585 °C.s^−1^) as a function of its length is linear (Figure 5a). The presence of pores inside the electrode is highlighted in Figure 4b. However, this presence does not significantly impact the electrical continuity of the electrode (longest length tested = 1 m). The theoretical behaviors calculated using Equation (2) for two different mono-electrode fibers presenting the same resistivity values as that of the AA 2024 alloy used to produce the preform (in red) or melted and then cooled down at 585 °C.s^−1^ (in blue) are also represented in Figure 5a. The experimental values (in black) are always superior to the calculated ones. The resistance for a 1 m long fiber is ten times that of the resistance of the AA 2024 alloy and three times that of the resistance of the melted AA 2024 alloy cooled down as the AA 2024 electrode was drawn (585 °C.s^−1^). An explanation could be made regarding the modification of the alloy microstructure during solidification.

The resistivity values of the mono-electrode fibers with various cooling rates and lengths from 3 to 5 cm are presented in Figure 5b. When the CR is 585 °C.s^−1^, the resistivity is around 10 times higher than the bulk pure copper resistivity (1.67 μΩ.cm [32]). In pure metals [33] and aluminum alloys [34], when grain size is reduced, resistivity is usually increased due to the increased number of grain boundaries. Decreasing the CR leads to a decrease in the resistivity of the mono-electrode fiber. Between 130 °C.s^−1^ and 290 °C.s^−1^, a minimum value around 6.9 × 10^−8^ Ω.m is reached. This behavior seems to agree with the decrease in the number of grain boundaries when the CR decreases.

### 3.4. Bi-Electrode Fibers and Plasma Generation

Given our knowledge of the drawing process to obtain a continuous electrode in a glass fiber, continuous bi-electrode fibers were also drawn for tip microplasma generation. According to Paschen’s law, there is a factor P.d (Pressure *×* Inter-electrode distance) for which the Breakdown Voltage (U_s_) needed to ignite a discharge in a gas or a mixture is the lowest [35]. Because this study was conducted in atmospheric pressure (plasma generation under air), the inter-electrode spacing had to be mastered. Its target value can be controlled either during step 1 or step 2 of the S&D process (Figure 1), i.e., by changing the preform design or the drawing stretching ratio.

**Figure 6:**
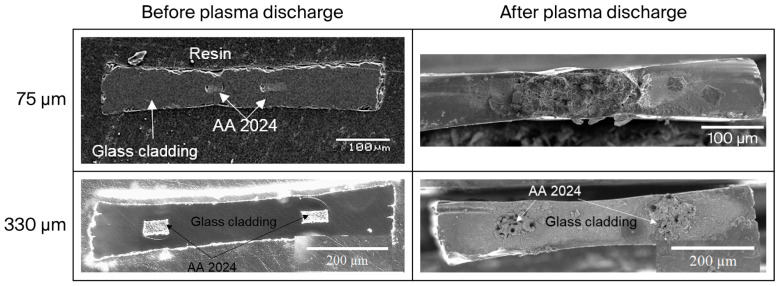
SEM pictures of the front cross-section of the bi-electrode fibers with an inter-electrode spacing of 75 or 330 µm before and after plasma discharge.

Two inter-electrode spacing values (75 µm and 330 µm) were chosen to investigate the effect of the inter-electrode spacing on the discharge characteristics and to maintain the voltage at relatively low value to generate the discharge. The experimental U_s_ values determined for both geometries were 0.9–1.1 kV and 1.5–2.2 kV, respectively. Figure 6 presents the front cross-sections of the bi-electrode fibers with an inter-electrode spacing of 75 or 330 µm before and after the plasma discharge.

First, the plasma discharge behavior is extremely different for the two bi-electrode fibers. The plasma stops after half an hour of discharge when the inter-electrode spacing is the smallest (75 µm). The control of the front cross-section after the 30 min plasma discharge reveals the presence of a continuous layer covering the two electrodes and the glass cladding interspacing. The electrical shortcuts are attributed to the formation of this layer, which prevents the plasma discharge.

Secondly, a wider 330 µm inter-electrode spacing allows for a continuous, electrically induced plasma discharge lasting for at least 6 h (at which point the experiment was voluntarily stopped). During the experiments, it was necessary to progressively increase the applied voltage from 1.5 kV at the beginning of the discharge (t = 0 h) to 2.5 kV before stopping (t = 6 h) to maintain the plasma discharge. Observation of the front cross-section after a 3 h plasma discharge reveals the presence of layers located on top of each electrode tip with no connection between the two electrodes (Figure 6).

### 3.5. Plasma Analysis

**Figure 7:**
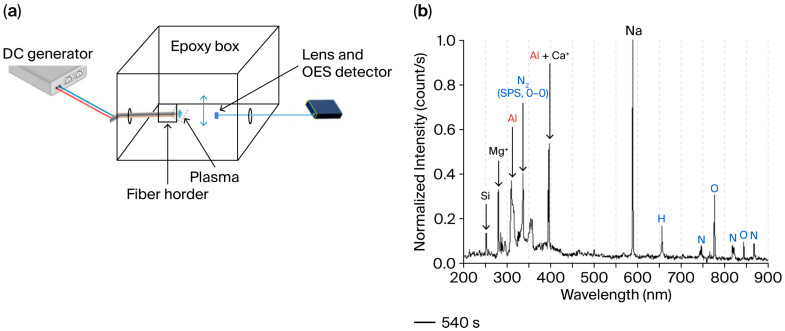
(**a**) Schematic representation of the experimental setup. (**b**) Example of OE spectrum recorded after 540 s of plasma discharge in air (inter-electrode spacing: 75 µm).

Optical emission spectra (OESs) were recorded during the plasma discharge in air. The database LIBS NIST was used to identify the main lines related to atomic relaxations [36]. Figure 7 presents the experimental setup mounted in a box for safety reasons (box volume 7.7 L ~30 × 16 × 16 cm^3^, Figure 7a. Figure 7b shows an example of an OES recorded after 540 s of plasma discharge in air. Line and band wavelength positions enabled the identification of the atoms and molecules deriving from the air, the glass and the electrodes. The line centered on 251.9 nm corresponds to the transition of a neutral Si (4s (^3^P^o^) → 3p (^3^P)). The line centered on 279.8 nm corresponds to a radiative transition from a singly ionized Mg^+^ (3p (^2^P^o^) → 3s (^2^S)) [36,37]. The line centered on 309.4 nm corresponds to the relaxation 3d (^2^D_3_) → 3p (^2^P^o^) of atomic Al [38]. As mentioned, the lines of Al and Ca^+^ overlap with each other between 393 and 397 nm due to the spectral resolution of the spectrometer. Fortunately, Al and Ca^+^ signals can be separated since Al also has two peaks centered at 394.4 nm and 396.2 nm, corresponding to the 4s (^2^S) → 3p (^2^P^o^) transition [36], while Ca^+^ has two lines centered at 393.3 and 396.8 nm, corresponding to the transition 4p (^2^P^o^) → 4s (^2^S) [39]. The line centered at 589.1 nm corresponds to the atomic relaxation of Na (transition 3p (^2^P^o^) → 3s (^2^S)) [40].

The other emission bands recorded originate from the elements of the air. The following emission wavelengths were chosen for their high intensity yield. Atomic N can be observed from the 868.2 nm line corresponding to the relaxation 3p (^4^D^o^) → 3s (^4^P) [41]. The 656.3 nm emission line corresponds to the relaxation 3d (^2^D) → 2p (^2^P^o^) of H [42], while the 777.2 nm emission line corresponds to the relaxation 3p (^5^P) → 3s (^5^S^o^) of atomic O [41]. The N_2_ Second Positive System (SPS) emission bands are located between 290 nm and 500 nm. They involve N_2_ relaxing through the electronic transition C^3^Π_u_ → B^3^Π_g_. Each electronic level can be associated with multiple vibrational levels. These vibrational levels are indexed using a vibrational quantum number (*v*). The 336.3 nm emission corresponds to the transition of N_2_ between the lowest vibrational state of both electronic states: C^3^Π_u_ (*v* = 0) → B^3^Π_g_ (*v* = 0). This line labeled N_2_ (0–0) is usually studied since it represents transition between fundamental electronic levels [43].

### 3.6. Bi-Electrode Fiber Behavior During Plasma Discharge in Air

**Figure 8:**
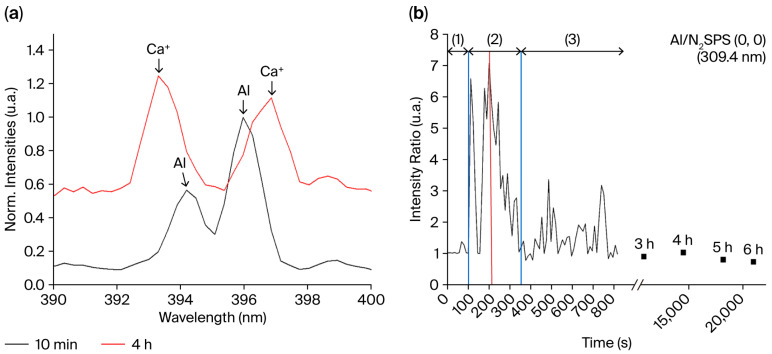
(**a**) Focus on the 390 to 400 nm OES region (inter-electrode spacing: 330 µm). Spectra obtained after 10 min (600 s) and 4 h (14,400 s) of plasma discharge in air. (**b**) Variation in the intensity ratio of the 309.4 nm emission line corresponding to Al and the 336.3 nm emission line corresponding to N_2_ SPS (0–0) as a function of time. The red line indicates the localization of the maximal intensity value.

As previously mentioned, the emission line of Al (element deriving from the AA 2024 electrode alloy) at 394.4 nm overlaps with the emission line of Ca^+^ (element deriving from the glass) at 393.3 nm, while the emission line of Al at 396.2 nm overlaps with the emission line of Ca^+^ at 396.8 nm. Figure 8a focuses on the 390–400 nm area, i.e., where the emission lines of Al and Ca^+^ are localized. At the beginning, when the plasma discharge starts, only the Al emission is observed (illustrated with the OE spectrum recorded at 10 min). The emission lines of Al and Ca^+^ are later present and overlap. Finally (illustrated with the OE spectrum recorded at 4 h) and until the latest recording, the Ca^+^ emission lines remain prevalent. To elucidate the phenomena occurring on the electrode tips, the evolution of the ratio between the intensity of the specific emission line of Al at 309.4 nm and the 336.3 nm emission line corresponding to N_2_ SPS (0–0) as a function of the plasma generation duration is presented in Figure 8b. The signal recorded appears to be noisy. This illustrates the main issue of OES analysis, which is that the analyzed plasma discharge turns on and off repeatedly. However, the evolution of the Al/N_2_ ratio can be divided into three steps: before 100 s, between 100 and 350 s and after 350 s. The red line indicates the localization of the maximum intensity (~220 s). Before 100 s, step (1) displays no emission as the applied voltage U is inferior to the U_s_ of the air. Step (2) highlights an emission increase as U reaches 1.5 kV. The peak intensity increases significantly from 100 s to 220 s, where a maximal value is achieved (around the red line). This phenomenon displays the sputtering of the AA 2024 electrodes as the quantity of Al present in the glass is negligible. Then, the emission presents a gradual decrease between 220 s and 350 s. During step 3, the emission appears to decrease slowly over time before stabilizing. At this step, precautions should be taken since the signal of Al overlaps with the signal of Ca. The signal of Al can be overestimated after 716 s. With the exception of a sudden increase in applied voltage, no significant evolution occurs between 800 s and the end of the experiment at 21,600 s (6 h).

**Figure 9:**
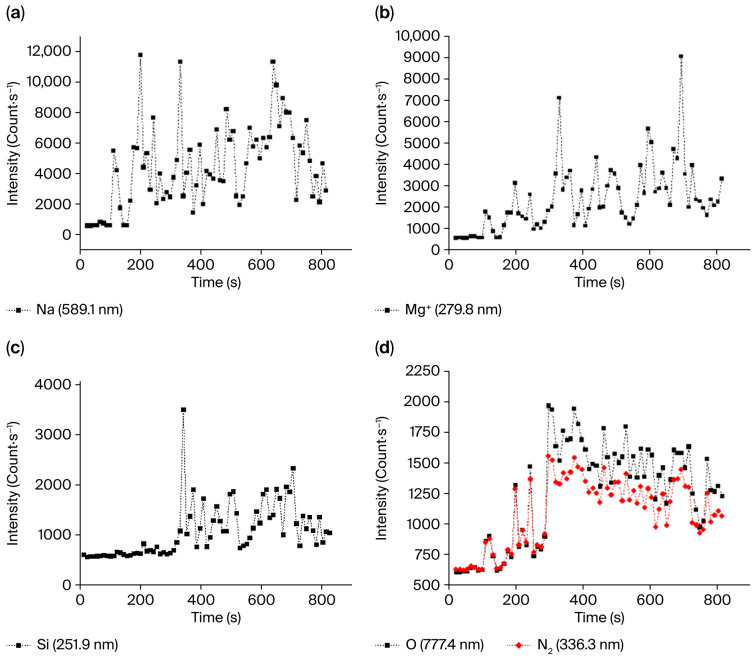
Variation in the peak intensity as a function of time: (**a**) 589.1 nm emission line corresponding to Na relaxation. (**b**) 279.8 nm emission line corresponding to Mg^+^ relaxation. (**c**) 251.9 nm emission line corresponding to Si relaxation. (**d**) 336.3 nm emission band corresponding to N_2_ (SPS) (0-0) relaxation (in black) and 777.2 nm emission line (in red) corresponding to O relaxation.

Figure 9 presents the intensity evolution as a function of time for the Na (589.1 nm), Mg^+^ (279.8 nm), Si (251.9 nm), N_2_ SPS (0–0) (336.3 nm, in black) and O_2_ (777.2 nm) relaxation peaks. The tendency for Na (Figure 9a) is a steady increase from 100 s to 700 s up to the point where the drop occurs. A similar behavior is observed for Mg^+^ (Figure 9b). The intensity of Si, mainly present in the glass cladding, starts to increase at around 300 s. This increase occurs around 150 s later than that of all the other elements. Rapid stabilization of the intensity is observed (Figure 9c). Emissions of N_2_ and O (Figure 9d in black and red, respectively), the primary components of the air, increase steadily between 100 s and 300 s, where they reach their maximum before decreasing steadily with time and then stabilizing.

### 3.7. Bi-Electrode Fibers Used as Gas Sensor

Tobacco fumes were used in this study to test the ability to detect molecules of interest (namely hydrocarbons) using a complex mixture of chemical compounds bound to aerosol particles or free in a gas phase [44]. The main chemical components are tobacco alkaloids, N-nitrosamines, polycyclic aromatic hydrocarbons, volatile compounds (including SO_2_, NO_2_, H_2_S…), aromatic amines, heterocyclic amines, as well as traces of heavy metals such as Cd, Co, Cr, Sb, Tl, Hg, Pb and As [45]. The total mass of components (sum of the emitted mass of all the above-mentioned components per cigarette) present in the smoke is estimated to be around 50 mg [46]. In the tests, 1 g of tobacco was weighed and placed in the chamber (Figure 7a) below the tip of the multimaterial fiber, where the microplasma generation took place at a distance close to 5 cm. Once the tobacco was ignited, the recording of OE spectra commenced. The hydrocarbon species density in the gas is estimated to be around 0.01 mg/cm^3^ in the closed epoxy chamber when all the tobacco is consumed.

**Figure 10:**
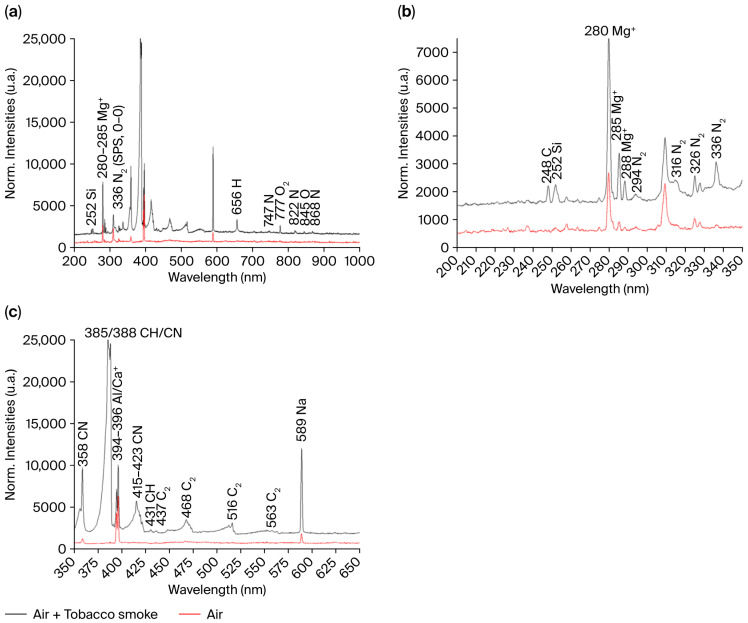
(**a**) OE spectra of air (in red) and air + tobacco fumes (in dark) recorded between 200 and 1000 nm after 300 s. (**b**) Zoom view between 200 and 350 nm. The emission line of C is visible for the air + tobacco fumes spectrum. (**c**) Zoom view between 350 and 650 nm. The Swan emission bands of C_2_ are visible as well as the band emissions of CN for the air + tobacco fumes spectrum.

Figure 10a shows the OE spectra recorded after 300 s when only air (in red) and air + tobacco fumes (in dark) are in the chamber. Emission bands corresponding to dicarbon radicals (C_2_ Swan bands) and carbon mono- and di-oxides are prevalent in the 300–600 nm region [47]. Both spectra present the band emissions of N_2_ and O_2_ as well as the line emissions of N, H, Si, Ca^+^, Na, Al and Mg^+^.

The zoom between 200 and 350 nm (Figure 10b) reveals the presence of a new emission line at 248 nm corresponding to the relaxation of neutral C (3s (^1^P°) → 2p^2^ (^1^S)). The presence of excited carbon is consistent with the carbon-rich nature of tobacco smoke. The zoom between 350 and 550 nm (Figure 10c) is more informative. When fumes are in the chamber, a small band at 431 nm attributed to a CH emission band is observed. The bands centered around 436, 468, 516 and 563 nm correspond to the emission of dicarbon “Swan” bands (C_2_), transiting from an upper to a lower state (d^3^Π_g_ → a^3^Π_u_). Transitions between two electronic states can be ordered following the difference in vibrational quantum numbers during the radiative transition between the upper and lower states $\left(∆v=v^{\prime }-v^{\prime \prime }\right)$. The 436, 468, 516 and 563 nm emissions correspond to ∆v= +2, +1, 0, −1 transitions, respectively. The presence of these bands indicates that the hydrocarbons contained in the tobacco smoke are decomposed. The bands centered around 358, 385–388 and 415 to 421 nm correspond to CN or some radiative transitions in N_2_ (SPS), CN/CH or some transitions in N_2_^+^ (FNS) and CN or some transitions in N_2_^+^ (FNS), respectively.

## 4. Discussion

### 4.1. S&D Parameters and Optimization of Electrical Properties of Multimaterial Fibers

During drawing (T_drawing_ = 800 °C), diffusion phenomena can occur at the interfaces between the metallic electrodes and the glass. The comparison of the DSC thermographs obtained for the AA 2024 alloy and the multimaterial fiber (Figure 2) shows no significant difference in the alloy melting temperature and dissolution of Al-Cu compounds. This result validates that the global composition of the AA 2024 alloy remains unchanged during the drawing process. If diffusion phenomena occur, they should be localized at the interfaces with the glass. Additional characterizations (EDS-STEM, nano-Auger…) are necessary to assess these diffusion mechanisms. From this statement, the variation in the resistivity of the mono-electrode fibers can be assumed to be strictly dependent on the drawing parameters, i.e., the microstructure of the electrode after solidification.

The small difference between the expected (based on the preform dimension) and the experimental fiber ratio W/T (4.12 and 3.94, respectively) could be explained by the rectangular opening in the heating ring of the drawing tower. It induces an asymmetric thermal gradient between the center of the preform and its edges during the draw, which prevents the draw from being truly homothetic. The sharp thermal profile of an inductive draw tower furnace, where the highest temperature is applied to a very thin area and then decreases rapidly along the vertical (z) axis, helps to preserve the preform geometry [48]. The use of an alloy with a high T_M_ helps to preserve its initial shape during drawing by decreasing the volume of drawn melt. The high viscosity of the glass cladding, combined with the moderate pressure from the melt, helps to prevent deformations at the glass–metal interface, which improves formability [8]. Metals with lower T_M_ form heavier melts that are not easily contained [8].

The addition of Cu and Mg to aluminum in the AA 2024 affects its viscosity in the liquid state (>T_M_) during drawing. In the literature, the viscosity (η) of aluminum increases while its fluidity decreases with the concentration of alloying elements [49]. The linear thermal expansion coefficient (α) decreases from 23.2 to 23.8 × 10^−6^ °C for pure aluminum to 22.8–23.1 × 10^−6^ °C for AA 2024 at 20 °C [50]. This helps to reduce the thermal shrinkage that happens during solidification and to prevent discontinuities in the electrodes and at the interfaces with the glass. The use of AA 2024 instead of pure Al shows a significant improvement of the electrode continuity in the fiber and helps to produce the longest conductive fibers.

Depending on the drawing parameters, the CR of the studied fiber varies from 130 °C.s^−1^ (V_drum_ = 2 m.min^−1^) to 585 °C.s^−1^ (V_drum_ = 9 m.min^−1^). Electrode microstructure changes occur during the thermal drawing since solidification mechanisms are impacted by the CR (to be published). The interface density increases with the solidification rate (increase in the number of grain boundaries with the reduction in grain size and interphase boundaries between the aluminum grains and the intermetallic Cu-Mg rich-phases). The interfaces act as traps that hinder electronic mobility, increasing the electrical resistivity of the AA 2024 alloy [51,52,53]. The presence of grain boundaries in the aluminum-rich phases correlates with the presence of large grains of Al_2_Cu and Al_2_CuMg phases (in white Figure 4b), which impacts the resistivity values with the change in the CR. The occurrence of defects in cast alloys is reported to be inversely proportional to the CR [53], while the increase in the porosity density drastically increases the resistivity ρ of aluminum alloys [54]. In this study, the presence of porosities (Figure 4b) and an increase in ρ with the CR (Figure 5b) are observed. To improve electrical properties, an external pressure could be applied during solidification to reduce the porosity density, as shown for pure copper [55]. Unfortunately, this solution is not possible in our fiber-drawing tower. Based on this study result, the best compromise to obtain better electrical properties for the AA2024 electrodes is to adapt process parameters to have CR values in the range 200–300 °C.s^−1^.

### 4.2. Microplasma Discharge

According to the classical Townsend mechanism, electrical breakdown occurs in gas when the kinetic energy of the electrons exceeds the threshold for ionization, allowing for the formation of ions via the electron-impact phenomenon [56]. The Paschen law shows that the lowest discharge voltage *U_s_* can be achieved by choosing a P × d ranging from 0.4 to 10 Torr.cm. With the bi-electrode fiber configuration of this study (Figure 3), the discharge is coplanar, and the P × d factor is about 5.6 Torr.cm (P = 1 atm = 760 Torr and d = 74.10^−4^ cm, respectively) and 25.1 Torr.cm (d = 330.10^−4^ cm). The U_s_ values are below 1 kV for a P × d around 5 Torr.cm and between 1 and 2 kV for a P × d between 10 and 20 Torr.cm for pure air plasma [25]. These values are coherent with the 0.9–1.1 kV for the 74 µm inter-spacing fiber and the 1.5–2.5 kV of the 330 µm inter-spacing fiber determined experimentally in this study. In contrast to a previous study with post-functionalization of the metallic electrodes [7], this study highlights the possibility of in situ functionalization at the tip of the electrodes (Figure 6, after plasma discharge). During the discharge, the layer formed is mainly composed of oxides. This corroborates the necessity to increase the applied voltage during the test. The growth of an oxide layer on the electrode tips is unavoidable during plasma discharge in the presence of oxygen (i.e., in air). It results from two phenomena: the deposition of the electrode material vapor or the direct oxidation of the electrode [57]. In the case of short inter-electrode spacing, bridging between the two growing layers at the electrode tips containing oxides induces the electrical shortcut. The growth mechanism of this layer can be studied with OES analyses. Even though alumina presents a high value of resistivity (10^14^ Ω.cm [58]), this value decreases with the temperature increase and in the presence of sodium oxide [59]. The elements of the glass cladding (Na, Si, Mg) are detected because of the damage to the glass surface during the generation of the plasma [60], especially when the electrode interspacing is low (Figure 7b).

It is difficult to discriminate the Al and Ca^+^ emissions because of the proximity of the Ca^+^ lines (at 393.3 nm and 396.8 nm, 4p (^2^P^o^) → 4s (^2^S)) with the ones of Al at 394.4 nm and 396.2 nm, 4s (^2^S) → 3p (^2^P^o^), as shown in Figure 8a. A shift between the 396.2 nm emission of Al and the 396.8 nm emission of Ca^+^ is also observable. The emission of Al decreases significantly until the emission lines of Ca^+^ overlap with it. The line intensity is linked to the concentration of Al in the plasma; therefore, its diminution can be linked to the oxide surface formation on the electrode tips that limits its evaporation. The decrease in the 309.4 nm line intensity, corresponding to the Al 3d (^2^D_3_) → 3p (^2^P^o^) relaxation, supports this theory of the in situ formation of an oxide layer covering the electrodes during plasma generation and its protective nature. A possible scenario for the oxide layer formation based on the OES analyses is as follows:

(1) Sputtering of the Al: emission increases (Figure 8b).
(2) Oxidation of the AA 2024 electrode surface due to the presence of oxygen in the plasma (Al emission line decrease, Figure 8b).
(3) Incorporation in the plasma of the glass cladding elements leading to the formation of the oxide layer. The triple junction effect enhances the interaction between the glass surrounding electrodes and the plasma because of the electric field amplification due to the free spaces between the glass and the electrodes [61]. This enhancement makes discharges more probable on the glass close to the electrodes and explains why most of the glass remains almost untouched by the discharge (see Figure 6, where the central part of the glass is not damaged).

The plasma is stabilized 150 s after the initial ignition at 100 s as the O_2_ and N_2_ emissions are stabilized around this time. At a fixed voltage, the band emissions of N_2_ and O_2_ decrease, reinforcing the hypothesis that the layer formed on the electrode surface is dielectric. The addition of a dielectric barrier on top of the electrodes decreases the amount of applied voltage that traverses the gas gap [26]. In order to maintain the discharge during plasma discharge, it is necessary to increase the voltage over time when the oxide layer entirely covers the electrodes as the oxide decreases the discharge power. In the case of the inter-electrode spacing of 330 µm, increasing U to 2.5 kV was enough to maintain the plasma. Despite an increase in Joule heating of the electrodes, no melting of the AA2024 alloy along the electrodes or deformation of the external ribbon glass fiber was observed.

It is also interesting to note that the N_2_^+^ and O_2_^+^ emissions are not visible in this spectrum. Therefore, the electron kinetic energy must be enough to ionize the neutral atoms and molecules composing the air, but insufficient for their relaxation to be visible. Overall, the absence of visible ionized species prevents an excessive increase in the gas temperature and allows the generation of a so-called low-gas temperature plasma [14].

### 4.3. Microplasma Gas Sensor

The new emission bands detected in the spectrum of the micro-plasma generated in air in the presence of tobacco fumes are identified as related to the relaxation of excited C_2_ molecules and CH/CN species (Figure 10). In the case of C_2_ molecules, these results are consistent with the research on C_2_ Swan bands as they are known to be produced from burning hydrocarbons [62]. Even though their building blocks are carbon atoms, they cannot be formed from CO_2_ [63]. Some of the proposed production pathways start from excited CO molecules via thermal decomposition [64] or the Boudouard mechanism [65] in a simple gas mixture. In the case of a complex gas mixture, the production pathways are not resolved.

The presence of a mixture of CH and CN is debatable as it is directly in the regions of relaxation transitions between radiative states of the N_2_ SPS (C^3^Π_u_ → B^3^Π_g_) and N_2_^+^ FNS (B^2^Σ_u_^+^ → X^2^Σ_g_^+^). These two systems are typically dominant in nitrogen-rich plasma in the 290–500 nm region. The main bands of each of these systems are not located at 388 nm, and the overlapping of their emission regions cannot explain the appearance of the massive 388 center band emission. The presence of excited CN in the microdischarge is validated as it is a prominent feature of plasma generated in active nitrogen and carbonaceous species. The formation of these species remains quite misunderstood as it depends on many competing pathways, even for simple gas mixtures [66]. It is dependent on gas composition: in a methane/nitrogen mixture, a reaction between C and N_2_ is the main pathway towards CN formation [67], while in an O_2_/N_2_/CO_2_ gas mixture, the N_2_ and CO reaction is predominant for CN formation [68].

**Figure 11:**
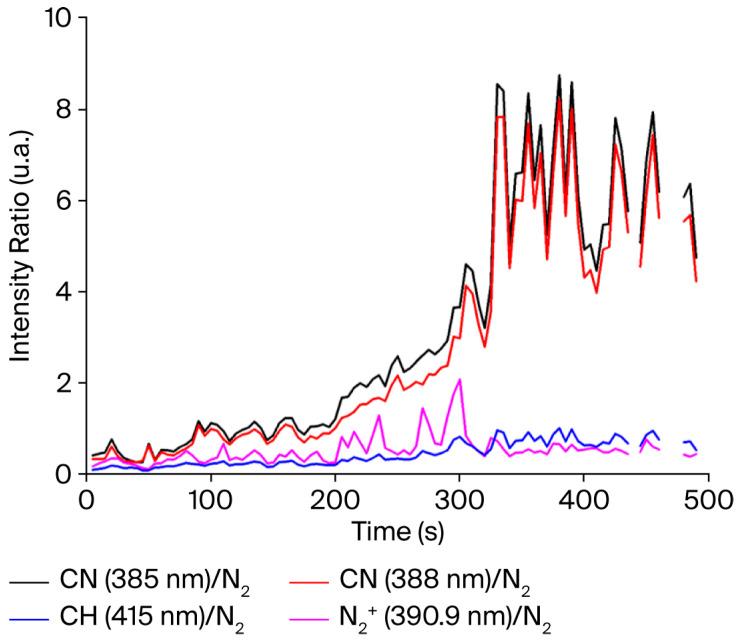
Example of the evolution of the CN/N_2_, CH/N_2_ and N_2_₊/N_2_ intensity ratios during the analysis of tobacco fumes.

The CN and CH species meet the same issue as the C_2_ molecules in terms of quantitative characterization. In order to compare the evolution of the spectral bands linked to hydrocarbon species in the discharge, the ratios of the band intensities centered at 385.5, 388.2 nm and 415.6 nm are divided by the intensity of the 337 nm N_2_ SPS (0,0) bands. Tracking the intensity ratio of the spectral bands of hydrocarbon species using N_2_ SPS (0,0) enables their contribution to the discharge to be monitored. Figure 11 presents the evolution of these ratios depending on the tobacco combustion time. The ratios of CH/N_2_ and N_2_^+^/N_2_ remain constant during the analyses (blue and pink, respectively, in Figure 11) when the CN/N_2_ ratios increase (dark and red). This shows that CN species are being formed and detected. The use of bi-electrode multimaterial fibers as a gas sensor is demonstrated.

The sensitivity of the set-up to gas species that are produced from burning hydrocarbons, as well as its ability to detect them, is evidenced in a closed space for the hydrocarbon species density in the gas estimated to be around 0.01 mg/cm^3^ (10 ppm). Such a sensor could be interesting for monitoring hydrocarbon emissions in isolated places (such as the exhaust of a car) and could help to reduce carbon emissions. The development of such new fibers is required with the development of Portable Emission Measurement Systems (PEMSs), which have transformed the landscape of emissions monitoring, providing real-time data on pollutant levels and allowing for on-road and in-field measurements. At the forefront of innovation, Micro-PEMSs enable nano-level precision in hydrocarbon emission measurement. These miniature devices—often equipped with advanced sensors and artificial intelligence—enable highly detailed monitoring processes in confined spaces as does the new generation of dual-electrode composite fibers developed in this study. However, challenges persist. Calibration, standardization and cost-effectiveness are hot topics of ongoing consideration [69].

## 5. Conclusions

In this study, multimaterial ribbon fibers composed of soda–lime silicate glass and AA 2024 electrodes were successfully elaborated via the S&D technique. The main conclusions are as follows:

(1) The selection of an aluminum alloy such as AA 2024, instead of a pure metal, maintains the continuity of the electrodes during thermal drawing. The viscosity increases and the fluidity decreases due to the presence of alloying elements (Cu and Mg for the AA 2024) relative to pure aluminum, leading to a significant reduction in the electrode discontinuity. The right choice of T_drawing_ is also essential to maintaining a high viscosity for the glass, thus preserving the preform shape (reducing dimensions) and containing the molten alloy.
(2) An understanding of the microstructural changes and porosity formation is essential to controlling the electrical resistivity value. The cooling rate (i.e., v_drum_ and T_drawing_) is the key parameter. With the chosen bi-electrode geometry and materials, the CR remains between 200 and 300 °C/s.
(3) The inter-electrode spacing value is critical for inducing the formation of a non-bridging layer of oxides between the electrodes during plasma generation. The in situ formation of an oxide layer on the tip of each electrode drastically increases the duration of the plasma emission without the need for additional post-functionalization of the electrode surface.
(4) Such a device (the multimaterial fiber developed in this study combined with an OES detector) can be used as a miniaturized gas detector for hydrocarbon emission detection. Proof of concept has only been demonstrated. The sensor properties still need to be finalised. Further work is required to build on the findings regarding the reproducibility of the sensitivity of such a system to different concentrations of harmful substances, or mixtures of them, for example.

## Figures and Tables

**Table 1:** Material compositions and physical properties (suppliers’ data).

Material	Composition (%at)	*T_g_* (±2 °C)	*T_x_* (±2 °C)	*T_M_* (±2 °C)	n@589 nm (±0.003)	*ρ* (10^−8^ Ω.m)	Density
Al-alloy AA 2024	96.0% Al; 2.0% Cu; 1.6% Mg; Traces (Si, Fe…)	-	-	662	-	3.95	2.71
Glass	72.7% SiO_2_; 12.0% Na_2_O; 6.9% CaO; 8.0% MgO; 0.4% Al_2_O_3_	562	826	-	1.517	-	2.48

## Data Availability

The data can be found on the website: https://entrepot.recherche.data.gouv.fr/dataverse/icmcb.
